# Optical Characteristics of MgAl_2_O_4_ Single Crystals Irradiated by 220 MeV Xe Ions

**DOI:** 10.3390/ma16196414

**Published:** 2023-09-26

**Authors:** Abdirash Akilbekov, Arseny Kiryakov, Guldar Baubekova, Gulnara Aralbayeva, Alma Dauletbekova, Aiman Akylbekova, Zhulduz Ospanova, Anatoli I. Popov

**Affiliations:** 1 L.N. Gumilyov Eurasian National University, Astana 010000, Kazakhstan; guldar_87@mail.ru (G.B.); agm_555@mail.ru (G.A.); alma_dauletbek@mail.ru (A.D.); aiman88_88@mail.ru (A.A.); zhulduz-ospan@mail.ru (Z.O.); 2 Ural Federal University, 21 Mira Str., 620002 Yekaterinburg, Russia; arseny.kiriakov@urfu.ru; 3 Institute of Solid State Physics, University of Latvia, 8 Kengaraga Str., 1586 Riga, Latvia; anatoli.popov@cfi.lu.lv

**Keywords:** single crystals MgAl_2_O_4_, absorption spectra, swift heavy ions, radiation defects, Raman spectra

## Abstract

In In this study, the optical properties of magnesium-aluminate spinel were examined after being irradiated with 220 MeV Xe ions. The research aimed to simulate the impact of nuclear fuel fission fragments on the material. The following measurements were taken during the experiments: transmission spectra in the IR region (190–7000) nm, optical absorption spectra in the range (1.2–6.5) eV, and Raman spectra were measured along the depth of ion penetration from the surface to 30 µm. A peak with a broad shape at approximately 5.3 eV can be observed in the optical absorption spectrum of irradiated spinel crystals. This band is linked to the electronic color centers of F^+^ and F. Meanwhile, the band with a maximum at ~(3–4) eV is attributed to hole color centers. Apart from the typical Raman modes of an unirradiated crystal, additional modes, A1g* (720 cm^−1^), and Eg* (385 cm^−1^), manifested mainly as an asymmetric shoulder of the main Eg mode, are also observed. In addition, the Raman spectroscopy method showed that the greatest disordering of crystallinity occurs in the near-surface layer up to 4 μm thick. At the same time, Raman scattering spectroscopy is sensitive to structural changes almost up to the simulated value of the modified layer, which is an excellent express method for certifying the structural properties of crystals modified by swift heavy ions.

## 1. Introduction

Some of the dielectric materials, wide-gap oxides, nitrides, perovskites, and diamonds have the highest radiation resistance. Specifically for thermonuclear programs, MgO, Al_2_O_3_, MgAl_2_O_4_, BeO, AlN, Si_3_N_4_ diamonds, and a few others are getting special attention [1,2,3,4,5,6,7,8,9,10,11]. Several objects, such as MgO and Al_2_O_3_, have been deemed model objects by researchers [12,13,14,15]. However, numerous other objects have significant importance in practical applications. Out of all the materials, the magnesium-aluminate spinel (MgAl_2_O_4_) is given particular focus due to its remarkable radiation resistance (as noted in references [16,17,18,19,20,21,22,23,24,25,26]). Choosing MgAl_2_O_4_ spinel as a matrix for transmuting actinides by capturing neutrons in nuclear reactors [27] is crucial. It is also a suitable matrix for storing radioactive waste. Additionally, the rapid advancement of photonics and electronics necessitates the creation of novel functional materials possessing unique characteristics such as exceptional radiation resistance, transparency across a broad spectral range, and robust thermal stability. MgAl_2_O_4_ is a material that can be effectively doped with transition 3d elements and rare earth ions to achieve desired optical properties [25,26,27,28]. This material is being considered for use in various applications including laser media [29], crystal phosphors, 3D printing [30,31], and scintillators [32]. It is also being explored as a matrix for high-temperature fiber-optic sensors [33,34] and as a porous material for moisture sensors [35]. Magnesium-aluminum spinel is used as a substrate for growing thin films [36,37].

The dominant effect of radiation damage in these materials is radiation-induced absorption due to the formation of so-called color centers. Thermal annealing or optical bleaching can partially or completely remove radiation-induced absorption. Currently, we only have adequate knowledge about the structure and behavior of radiation defects, such as electronic F-type centers and hole V-type defects, in binary oxides like ionic MgO and partially covalent Al_2_O_3_, which are the structural components of MgAl_2_O_4_. When it comes to MgO and Al_2_O_3_, studies on thermal annealing have revealed that their kinetic properties are significantly influenced by the dose they receive. Specifically, the activation energies of these materials decrease as the dose increases, and the exponents are dependent on the activation energy. This phenomenon is referred to as the Meyer-Neldel rule and has been supported by extensive experimental data [38]. Meanwhile, the findings for MgAl_2_O_4_ exhibit contrasting behavior, potentially linked to the unique function of anti-site defects (ADs) [14,15]. and require more detailed study. The growth of complex oxides is characterized by the presence of cationic disordering, which leads to the formation of charged ADs defects. This is a significant feature of the process. Research is needed to understand how they affect the optical, luminescent, and radiation-induced properties. Information regarding threshold displacement energies and optical characteristics of point defects can be found in the literature sources [25,26,27]. Currently, there is a lack of organized data on radiation defects in MgAl_2_O_4_, particularly those caused by fast neutrons and high-energy heavy ions.

This study aims to examine the radiation-induced defects that occur in spinel crystals when exposed to fast heavy ions, specifically xenon with an energy level of 220 MeV. This will be accomplished using optical and Raman spectroscopic techniques.

## 2. Materials and Methods

**Figure 1:**
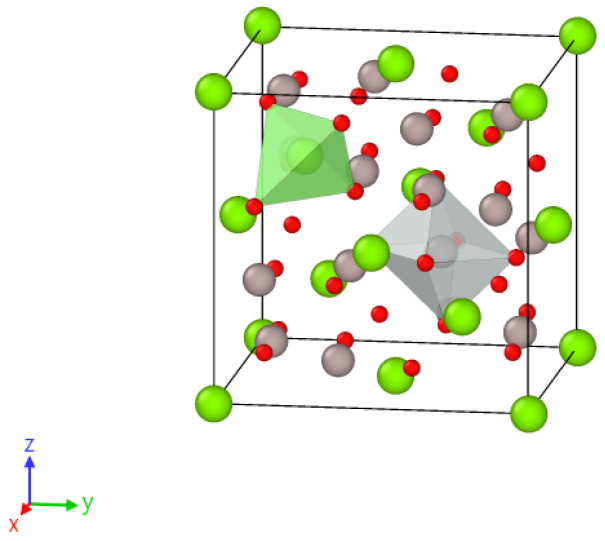
The elementary cubic cell of MgAl_2_O_4_. The color-coding scheme used is green for Mg atoms, gray for Al atoms, and red for O atoms. The positions of Mg and Al in tetragonal and octahedral positions are marked in green and red, respectively.

For this study we used optically transparent samples of MgAl_2_O_4_ spinel with an unbroken stoichiometry of 0.5 mm thickness. These samples were grown by the Czochralskii method and provided by the German company “ALINEASON”. The MgAl_2_O_4_ crystal lattice structure is a tightly packed cubic arrangement of negative oxygen ions and positive metal ions. It belongs to the space group of Fd3m. In the typical arrangement of spinel, Mg^2+^ ions are found in 1/8 of the tetrahedral positions with T_d_ symmetry (also known as the A site). Meanwhile, Al^3+^ ions occupy 1/2 of the octahedral positions with D_3d_ symmetry (the B site) (as shown in Figure 1). In partially reversed spinel crystals, trivalent metal ions of Al^3+^ can move to tetra-positions instead of ions of divalent Mg^2+^; similarly, Mg^2+^ can move to octa-positions instead of Al^3+^, creating anti-site defects.

**Figure 2:**
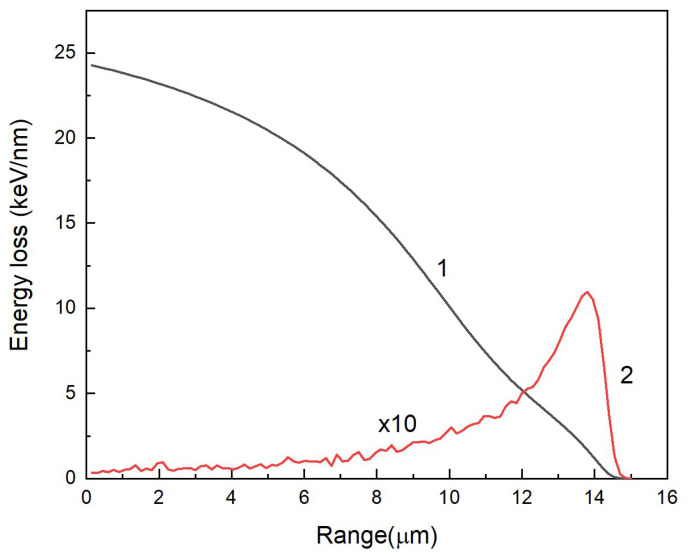
Electronic (S_e_) and nuclear (S_n_) ion energy losses of 220 MeV Xe ions according to SRIM 2013 [39].

The samples under investigation were exposed to high-energy heavy Xe ions (220 MeV) at room temperature, perpendicular to the (111) plane at cyclotron DS-60 in Astana, Kazakhstan. The fluence range was from 10^10^ to 10^14^ cm^−2^. After irradiation, the samples were placed in zip-lock bags and stored in a dark place for one month. The main parameters of Xe ions in MgAl_2_O_4_ crystals were calculated using the SRIM 2013 code [39] and are presented in Table 1 and Figure 2.

The ratio of S_e_/S_n_ = 328 i.e., specific ionization losses are dominant, and the main mechanism of defect creation is related to electronic excitations. Nuclear (elastic) energy losses begin to dominate at the end of the ion range. Specific ionizing energy loss of 220 MeV Xe ions in the probed subsurface layer is 24.3 keV/nm which is significantly higher than the threshold of latent track formation in MgAl_2_O_4_, ~7.5 keV/nm [40,41,42] Therefore the possible effects of ion track-associated radiation damage should be taken into account.

Raman spectra (RS) were recorded using a LabRam HR800 Evolution confocal spectrometer (Horiba, Japan), and excitation was performed with a 514 nm laser. It should be noted that the main advantage of the confocal measurement method is the possibility of focusing the excitation light beam and recording emission exclusively in the near-surface layer (~2 μm) of the sample with a sufficiently high spatial resolution. This makes it possible to ignore radiation defects formed through the elastic scattering channel, considering only defects formed because of the relaxation of electronic excitations. In addition, because of the localization of the excitation light in the irradiated region of the sample, the contribution of the non-irradiated part of the crystal and impurities is minimized [43].

Optical absorption (OA) spectra in the range (190–1100) nm of the virgin and irradiated samples were measured on a Lambda 35 spectrophotometer (PerkinElmer, Waltham, MA, USA). IR spectroscopy was performed on a Shimadzu IR-Prestige-21Fourier spectrophotometer, Japan, (2000–8000) nm.

## 3. Results

Optical transmittance is an important characteristic of functional crystals. For alumina-magnesium spinel, the optical transparency window lies in the range from vacuum UV λ ~150 nm to mid-IR λ ~6.5 μm. The optical transmittance in the virgin investigated crystals is in the indicated ranges. The transmission spectrum corresponds to the theoretical values of 88%. Absorption in the UV range is mainly due to zone transitions. The upper valence band consists mainly of 2p O states and is hybridized with the 3s-orbitals of Mg and the 3p-orbitals of Al. The conduction band includes compounds in both Mg 3s and Al 3p states [44].

Absorption in the long-wavelength spectral part of the optical transmittance is mainly due to the vibronic component. Here, the main contribution is made by the Mg-O and Al-O vibrations, forming the long-wavelength absorption edge.

**Figure 3:**
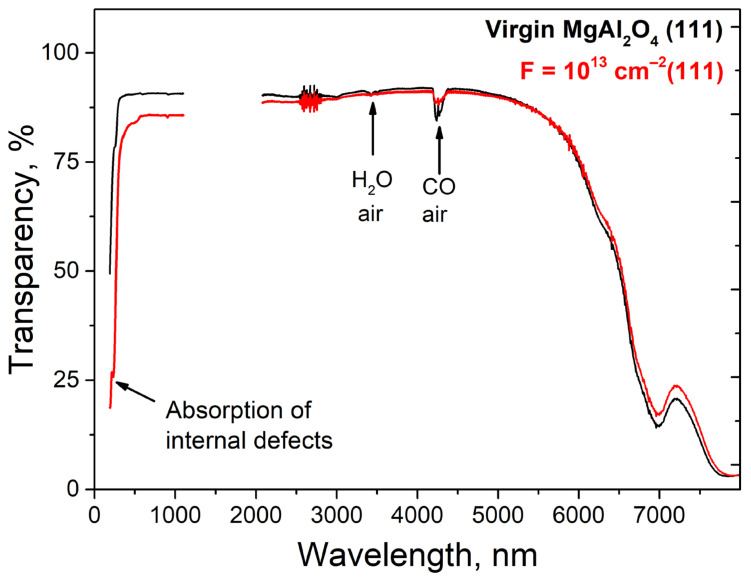
Optical transmission spectra of MgAl_2_O_4_ crystals before (black) and after (red) irradiation with 220 MeV xenon ions.

The optical transmission spectra of the initial sample, as well as the sample irradiated with the maximum dose, register signals associated with vibrations of water molecules H_2_O as well as CO present in the air, as shown in Figure 3.

Upon ion irradiation, there is a decrease in optical transparency in the UV spectral range caused by the creation of intrinsic defects in the anionic sublattice. In addition, a slight brightening in the long wavelength spectral region is observed, which appears to be due to the interaction of the cationic component of the matrix with the oxygen backbone. Since the UV spectral region showed increased sensitivity to SHI (Swift Heavy Ions), the optical absorption spectra of the visible and UV ranges were additionally analyzed. The vibrational spectra have also been studied in more detail using Raman spectroscopy.

**Figure 4:**
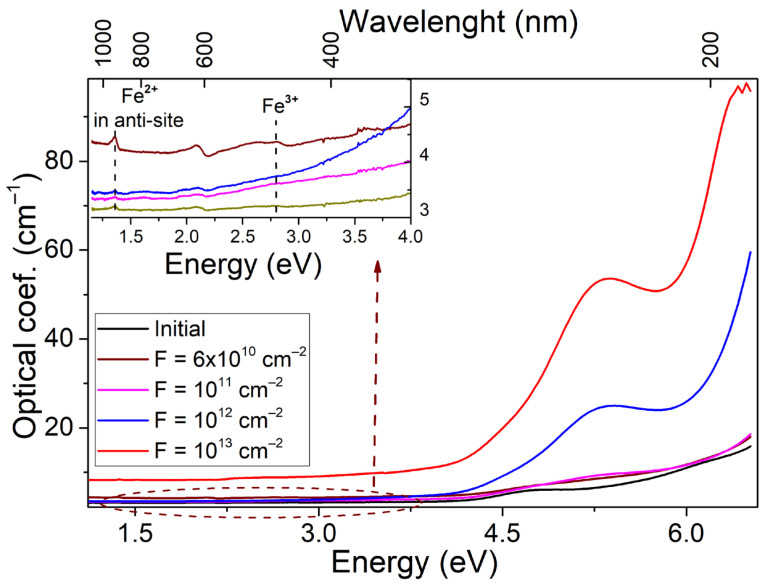
Raman spectra of spinel single crystals irradiated with 220 MeV Xe ions to a fluence of 10^13^ ions/cm^2^ as a function of depth.

The initial and irradiated single crystals differ slightly in transparency in the visible spectral range. The main contribution to the change in optical characteristics occurs in the UV spectral region, as shown in Figure 4. The states associated with the oxygen sublattice are mostly responsible for the above region. Thus, in numerous works on the interaction of spinel crystals and ceramics with high-energy radiation, it was found that the UV spectral region is modified because of the formation of optically active defects of vacancy type. According to the literature [25,26,45,46,47,48,49,50,51,52], the broad complex radiation-induced absorption band with a peak around 5.3 eV is mainly due to the electronic color centers of F^+^ and 4.75 eV F centers, while the hole color centers (V) are responsible for the optical absorption at ~3–4 eV.

A weak signal of optically active Fe^3+^ ions corresponding to the 6A1g→4Eg (2.7 eV) and 5T2→5E transitions from Fe^2+^ localized in octahedral nodes of the lattice is also registered. In both cases, the signal intensities are extremely small. Optical absorption shows how sensitive the spinel anion sublattice is to ultrahigh energy ion irradiation. At the same time, the calculated phonon curve as well as changes in the long-wavelength spectral region of optical transmittance indicate that in the process of SHI irradiation, there is an effective interaction in the cationic sublattice. One of the known types of such interaction is the formation of a special type of defect in the spinel matrix. Due to the close ionic radius of Mg^2+^ and Al^3+^ cations, their partial substitution in the matrix with the formation of anti-site defects Mg^2+^|_Al3+_ и Al^3+^|_Mg2+_ is possible. Moreover, anti-site defects in aluminum are formed with a large dominance. Table 2 shows the designation and nature of the observed bands in the optical absorption spectrum of the investigated objects.

The optical absorption spectra were decomposed into known components, revealing that the origin crystals have low concentrations of F and F^+^ type centers. There is an extra band with a peak at 6 eV in the UV spectral part. The indicated type of centers is apparently due to intrinsic lattice defects. These imperfections were created due to the growth of crystals. The absorption increases above 6.5 eV due to localized charge carrier states near the fundamental absorption edge.

**Figure 5:**
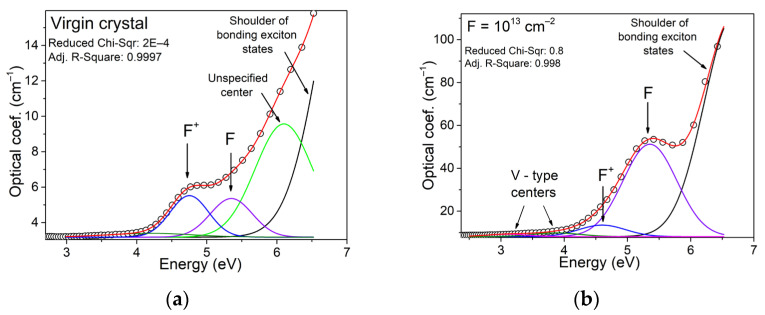
Decomposition of the optical absorption spectra of the original (**a**) and irradiated to a fluence of 10^13^ cm^−2^ (**b**) crystal. Open circles represent the original data, solid lines represent the results of deconvolution. The red line is the summed spectrum, the blue component is the contribution of optically active F^+^ centers, the violet component corresponds to the contribution of F centers, and the black component is responsible for the contribution of the excitonic component.

Irradiating of crystals by SHI significantly increases their optical absorption in the UV spectral region. The concentration of anion vacancies increases with the formation of F-type centers, as shown in Figure 5. Furthermore, there has been a shift towards the low-energy tail region of localized states. This process is likely due to the formation of the Urbach tail because of ion-induced disorder in the crystal lattice near the ion track. A study on modifying spinel crystals with accelerated electrons observed a similar pattern of changes in high-energy optical absorption [49]. The main difference between SHI and electron modification is the selectivity of absorption band formation, and the fluences required for significant changes in the modified layer’s structure. It is important to mention that the strength of the absorption bands for F and F^+^ centers, as described in the literature [49], can be approximated at 1. Table 3 shows that using SHI, we can estimate a linear upward trend at the 10 levels under logarithmic fluence scale conditions.

**Figure 6:**
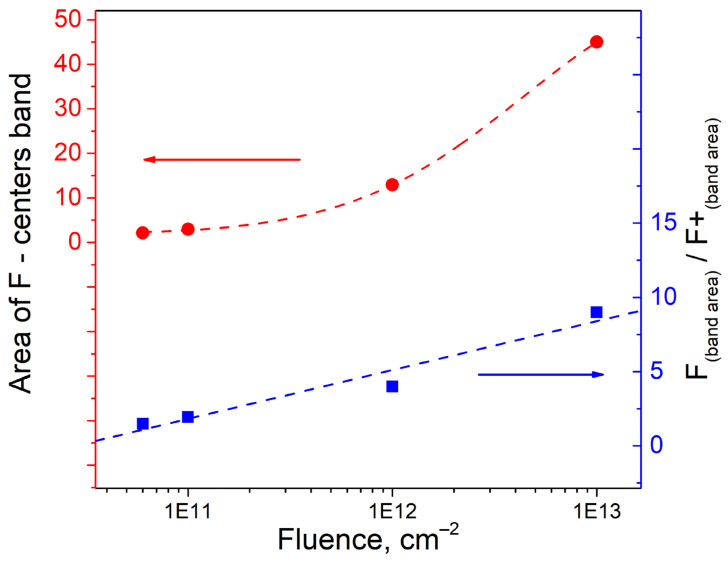
Integral area of F-type centers (red), and the ratio of the integral areas of F to F^+^ bands as a function of the fluence of accelerated ions (blue).

Extrapolating these results to higher fluences is likely to result in a nonlinear increase in the ratio of optically active centers, Figure 6.

The formation of anionic vacancies, whether from accelerated ions or electrons, results in the knock-out of an oxygen ion via a knock-out mechanism. In the case of electronic modification, F centers undergo strong ionization, leading to the formation of F^+^. In this instance, the electrons that are moving faster interact with the electrons that are trapped in the oxygen vacancy. When irradiated with high-energy ions, electron excitations are created. This creates high-energy electrons that move freely in the crystal. At the same time, the formed anionic defects require compensation by negative charge, and the local compensation is such that it is necessary to directly form F centers (a pair of trapped electrons). The probability of ionizing the F center under SHI is lower than the formation of anionic vacancies.

Magnesium anti-site defects are formed predominantly due to sufficient thermal stimulation, increasing the intensity of oscillations of the “breathing” mode of the oxygen octahedron. At the formation of a pair of anti-site defects of aluminum and magnesium, the local electroneutrality of the lattice is complied. Registration of defects of this type is usually difficult since pairs of such defects represent an electroneutral complex, which is not active for such sensitive methods as optical and ESR spectroscopy.

At the same time, a sufficient concentration of anti-site defects leads to corresponding distortions in the phonon spectrum. In Ref. [56] it was shown that stimulation of spinel ceramics with 10 MeV electrons allows the formation of additional anti-site defects. In the case of ion irradiation, the particle range is much lower, but the secondary collision cascades should generate cationic mixing.

**Figure 7:**
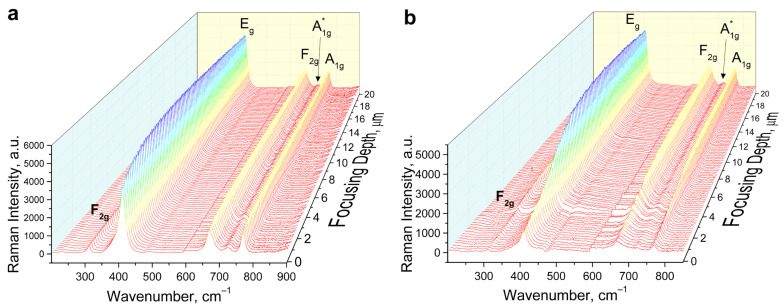
Raman spectra of spinel single crystals irradiated with 220 MeV Xe ions to a fluence of 10^13^ ions/cm^2^ as a function of depth. Non-irradiated side (**a**), irradiated side (**b**).

Figure 7 shows the results of Raman spectra recorded from different laser focusing depths on crystal (111) unirradiated side (Figure 7a) and irradiated side with a dose of 10^13^ cm^−2^ (Figure 7b). Characteristic vibrational modes F_2g_ (1) (312 cm^−1^) E_g_ (408 cm^−1^) F_2g_ (3) (670 cm^−1^) A_1g_ (768 cm^−1^) are registered. In addition to the characteristic Raman modes of an ideal crystal, additional modes, A_1g_* (720 cm^−1^), and E_g_* (385 cm^−1^), manifested mainly as an asymmetric shoulder of the main E_g_ mode, are also observed.

According to [39], the Raman peak at ~766 cm^−1^ is due to internal vibrations of MgO_4_ structural units, while the Raman peak at ~722 cm^−1^ is caused by the process of Mg-Al cation disorder, (i.e., formation of AlO_4_ structural units). Thus, cationic mixing occurs along the Xe ion pathway. The 408 cm^−1^ peak gradually broadens along the depth of ion penetration, indicating amorphization of the structure along the ion trajectory.

**Figure 8:**
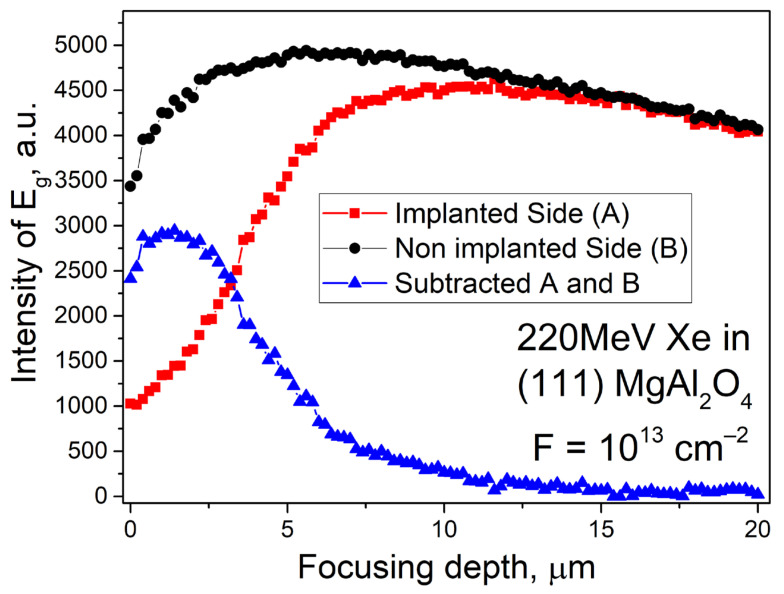
Intensity of the main vibrational mode E_g_ for the non-irradiated (black circles) and irradiated (red squares) sides. Blue triangles show the contribution of ion irradiation to the decrease in the relative intensity of the E_g_. mode.

Figure 8 shows the dependence of E_g_ mode intensity on depth. The non-irradiated side shows a stable intensity of the main Eg co-oscillatory mode. There is a decrease in the intensity of oscillations on the surface due to laser focusing errors, as well as a slow decrease in intensity at depths above 10 μm, which is due to the effect of light scattering. The intensity of E_g_ increases with increasing depth, reaching a maximum value of 13 µm which remains almost unchanged until the end of the xenon ion’s 14 μm range.

The non-irradiated side of the sample shows parasitic scattering effects of laser radiation that complicate Raman scattering pattern with changes in focusing depth. We subtracted the relative intensity curve of the main vibrational mode from the unirradiated side of the sample from that of the irradiated side (Figure 8, blue curve). The analysis of the difference curve shows that in the conditions of maximum energies of electronic losses (energy losses up to 20 keV/nm—typical for the thickness of the irradiated layer up to 4 μm), a maximum in the decrease of the intensity of the main vibrational mode is observed. This shows that the greatest amorphization of the crystal under ion irradiation is caused by accelerated ions in the near-surface layer. Further inhibition of the ion, due to the reduction of the transferred energy, generated fewer defects. This is accompanied by the growth of the main vibrational mode. The intensity of the vibrational mode starts to decrease noticeably at focusing depths of 13 µm and higher. This agrees with the calculation shown in Figure 2. Raman scattering spectroscopy enables non-destructive, rapid assessment of modified layer depth and vibrational characteristics affected by accelerated ions, which is also confirmed, for example, in [57,58,59].

## 4. Conclusions

MgAl_2_O_4_ crystals have exceptional radiation resistance, therefore spinel is chosen as a possible matrix for transmutation of actinides by neutron capture in nuclear reactors, as a matrix for storage of radioactive waste, and inert matrix of nuclear fuel. Other applications include photonics, electronics, crystal phosphors, and laser media in harsh radiation fields.

The optical characteristics of magnesium-aluminate spinel irradiated with fast heavy xenon ions modeling the effects of nuclear fuel fission fragments were investigated in this paper. The experiments measured transmission spectra in the IR region (240–12,500) cm^−1^, optical absorption spectra in the range (2–8) eV, and Raman spectra were measured along the depth of ion penetration from the surface to 30 μm. In the optical absorption spectrum of irradiated spinel crystals, a broad complex band of radiation-induced absorption with a peak around 5.3 eV is observed. This band is associated with electronic color centers of F^+^ and F type, while hole color centers are responsible for OA at ~(3–4) eV. In the near-infrared region, the irradiated crystal retains transparency. In addition to the characteristic Raman modes of an ideal crystal, additional modes, A1g* (720 cm^−1^), and E_g_* (385 cm^−1),^ manifested mainly as an asymmetric shoulder of the main E_g_ mode are also observed. The intensity of E_g_ Raman mode increases with the increasing depth of Raman spectra scanning, reaching a maximum of 13 μm which remains almost unchanged until the end of the xenon ion’s 14 μm range. The irradiation with 220 MeV ions leads to cation mixing along the ion pathway. The 408 cm^−1^ peak gradually broadens along the depth of ion penetration, indicating amorphization of the structure along the ion trajectory.

## Figures and Tables

**Table 1:** Parameters of 220 MeV Xe ions in MgAl_2_O_4_ crystals.

Ion & Energy, MeV	Fluence, cm^−2^	R_p_, μm	<S_e_>, keV/nm	<S_n_>, keV/nm
^132^Xe, 220	10^10^–10^14^	14.12	24.3	0.074

**Table 2:** Characterizations of defects in irradiated MgAl_2_O_4_.

Defect	Model	E_max,_ eV	Reference
V_c_ − with trapped carriers	V centers	3–4	[26,48]
F^+^	V_o_ + e	4.75	[53]
F	V_o_ + 2e	5.3	[53]
Fe^3+^ 6A1g→4Eg	Fe^3+^ in octahedral position	2.7	[54]
Fe^2+^ 5T2→5E	Fe^2+^ in octahedral position	1.2	[54]
Plasmon	Sample holder (Plasmon resonance on copper plate)	2.1	[55]

V_o_—oxygen (anion) vacancy); V_c_—cation vacancy.

**Table 3:** Estimating the area under the curve.

Fluence, cm^−2^	F-Centers Area	F^+^ Centers Area	F/F^+^
-	1.63	1.6	1.01
6 × 10^10^	2.15	1.45	1.48
1 × 10^11^	2.94	1.51	1.95
1 × 10^12^	12.92	3.23	4
1 × 10^13^	45	5	9
